# Correction: Exploring emotion recognition in patients with mild cognitive impairment and Alzheimer’s dementia undergoing a rehabilitation program emotion recognition in patients with dementia

**DOI:** 10.1371/journal.pone.0346658

**Published:** 2026-04-06

**Authors:** The PLOS One Staff

## Notice of Republication

This article was republished on March 13, 2026, to correct an error in the title that was introduced during the typesetting process. Erroneous text was inserted in the article title. The correct title is: Exploring emotion recognition in patients with mild cognitive impairment and Alzheimer’s dementia undergoing a rehabilitation program. The publisher apologizes for the errors. Please download this article again to view the correct version. The originally published, uncorrected article and the republished, corrected articles are provided here for reference.

## Supporting information

S1 File Originally published, uncorrected article. (PDF)

S2 File Republished, corrected article. (PDF)

## References

[1] Kamiya M, Osawa A, Otaka E, Kato K, Yoshimi T, Kagaya H, et al. Exploring emotion recognition in patients with mild cognitive impairment and Alzheimer’s dementia undergoing a rehabilitation program. PLoS One. 2025;20(4):e0322213. doi: 10.1371/journal.pone.0322213 40273125 PMC12021228

