# Correction: Exploring emotion recognition in patients with mild cognitive impairment and Alzheimer’s dementia undergoing a rehabilitation program

**DOI:** 10.1371/journal.pone.0347796

**Published:** 2026-04-21

**Authors:** Masaki Kamiya, Aiko Osawa, Eri Otaka, Kenji Kato, Tatsuya Yoshimi, Hitoshi Kagaya, Izumi Kondo

The phrase “Alzheimer’s dementia” should be “Alzheimer’s Disease” in the article title. The correct title is: Exploring Emotion Recognition in Patients with Mild Cognitive Impairment and Alzheimer’s Disease Undergoing a Rehabilitation Program. The correct citation is: Kamiya M, Osawa A, Otaka E, Kato K, Yoshimi T, Kagaya H, et al. (2025) Exploring emotion recognition in patients with mild cognitive impairment and Alzheimer’s Disease undergoing a rehabilitation program. PLoS ONE 20 (4): e0322213. https://doi.org/10.1371/journal.pone.0322213
