# Supplementary figures and images for: Exploring emotion recognition in patients with mild cognitive impairment and Alzheimer’s dementia undergoing a rehabilitation program
Source: PLoS One. 2025 Apr 24;20(4):e0322213. doi: 10.1371/journal.pone.0322213 (PMC12021228; doi:10.1371/journal.pone.0322213)

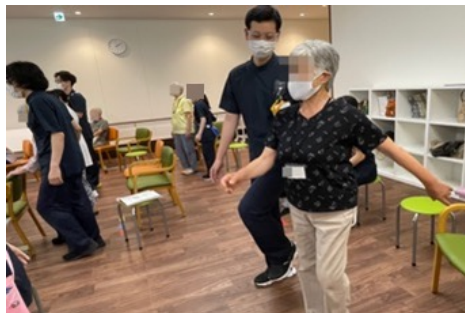

**Aerobic exercise**

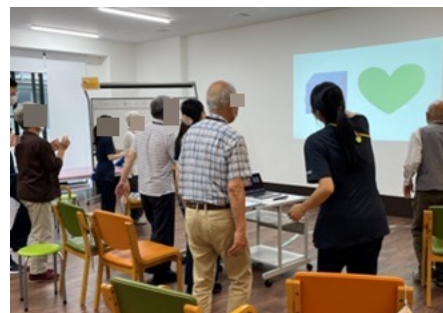

**Dual tasks**

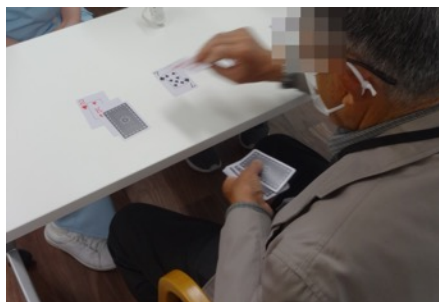

**Cognitive training**

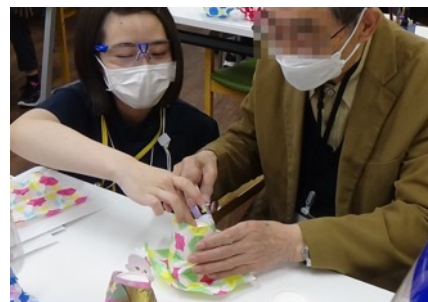

**Creative activities**

Supplement: S1 Fig — (PDF) [file pone.0322213.s001.pdf]

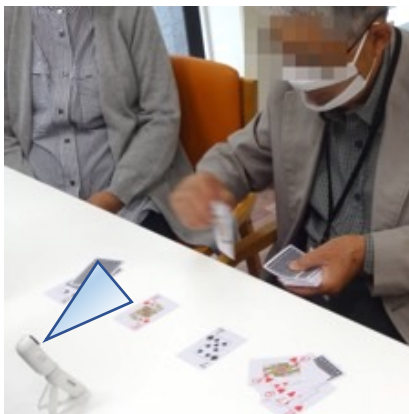

Supplement: S2 Fig — (PDF) [file pone.0322213.s002.pdf]
